# Profiling Anti-Apoptotic BCL-xL Protein Expression in Glioblastoma Tumorspheres

**DOI:** 10.3390/cancers12102853

**Published:** 2020-10-02

**Authors:** Deborah Fanfone, Ahmed Idbaih, Jade Mammi, Mathieu Gabut, Gabriel Ichim

**Affiliations:** 1 Cancer Research Centre of Lyon (CRCL) INSERM 1052, CNRS 5286, 69008 Lyon, France; Deborah.FANFONE@lyon.unicancer.fr (D.F.); Jade.MAMMI@lyon.unicancer.fr (J.M.); mathieu.gabut@inserm.fr (M.G.); 2 Cancer Cell Death Laboratory, Part of LabEx DEVweCAN, Cancer Initiation and Tumoral Cell Identity Department, CRCL, 69008 Lyon, France; 3 Université Claude Bernard Lyon I, 69100 Villeurbanne, France; 4 Sorbonne Université, INSERM, CNRS, UMR S 1127, Institut du Cerveau et de la Moelle épinière, ICM, 75013 Paris, France; ahmed.idbaih@aphp.fr; 5 AP-HP, Hôpitaux Universitaires La Pitié Salpêtrière-Charles Foix, Service de Neurologie 2-Mazarin, 75013 Paris, France; 6 Stemness in Gliomas Laboratory, Cancer Initiation and Tumoral Cell Identity Department, CRCL, 69008 Lyon, France

**Keywords:** glioblastoma, cancer stem cells, BCL-xL, apoptosis, BH3 mimetics, therapeutic opportunity

## Abstract

**Simple Summary:**

Glioblastoma is a fast-growing and very aggressive brain tumor. Its treatment is usually based on radiation and chemotherapy, which are inefficient owing to glioblastoma stem cells. Indeed, these cells are often resistant to therapy and are a source of tumor regrowth. Therefore, understanding how the survival of glioblastoma stem cells is regulated might unlock new therapeutic opportunities. We show here that certain glioblastoma cells, grown in specific conditions to favor glioblastoma stem cell proliferation, have a higher expression of BCL-xL, a protein preventing cancer cell death. This dependency on BCL-xL can be therapeutically targeted with specific BH3 mimetics drugs, designed to inhibit BCL-xL and thus induce efficient glioblastoma cell death. Overall, our study advocates for a better understanding of how to specifically target BCL-xL to trigger glioblastoma cell death.

**Abstract:**

Glioblastoma (GBM) is one of the cancers with the worst prognosis, despite huge efforts to understand its unusual heterogeneity and aggressiveness. This is mainly due to glioblastoma stem cells (GSCs), which are also responsible for the frequent tumor recurrence following surgery, chemotherapy or radiotherapy. In this study, we investigate the expression pattern of the anti-apoptotic BCL-xL protein in several GBM cell lines and the role it might play in GSC-enriched tumorspheres. We report that several GBM cell lines have an increased BCL-xL expression in tumorspheres compared to differentiated cells. Moreover, by artificially modulating BCL-xL expression, we unravel a correlation between BCL-xL and tumorsphere size. In addition, BCL-xL upregulation appears to sensitize GBM tumorspheres to newly developed BH3 mimetics, opening promising therapeutic perspectives for treating GBM patients.

## 1. Introduction

Glioblastoma (GBM) is the most frequent primary malignant and invasive adult brain tumor, with an extremely devastating prognosis since the five-year survival rate does not exceed five percent [1]. The current therapy (Stupp treatment) combines surgical resection of the primary tumor, radiotherapy and chemotherapy with the alkylating agent temozolomide (TMZ) [2]. However, despite these aggressive treatments, GBM invariably relapses as repeated surgery is rarely feasible and no consensual medical treatment is available, leading to tumor progression and patient death [3]. GBM recurrence can partially be explained by the important intra-tumoral cellular complexity of GBM tumors and by a high inter-tumoral heterogeneity [4]. Due to such pronounced cellular and molecular heterogeneity, GBM cells can adapt to selective pressures and changes in their microenvironment, leading to increased tumor aggressiveness and treatment resistance [5].

GBM tumors contain a small subset of cells with both stemness (self-renewing, proliferation, differentiation) and tumor-initiating properties, called glioblastoma stem cells (GSCs), driving tumor recurrence in GBM patients even after Stupp treatment [6,7]. Similarly to neural stem cells that have the capacity to form neurospheres *in vitro*, GSCs have the ability to form clonogenic structures, called tumorspheres, in serum-free non-adherent conditions [6,8]. These 3D cultures represent ideal models to unravel and test new therapeutic strategies to specifically target GSCs and prevent GBM progression [9].

Cancer cells must overcome several cellular stresses such as DNA damage, inflammation, oncogene activation, aberrant cell cycle progression and a harsh microenvironment that would normally trigger apoptosis in non-transformed cells [10]. Apoptosis, the most extensively studied form of programmed cell death, is essential in maintaining the homeostatic balance between cell proliferation and cell death. Apoptosis can be triggered by two major signaling pathways, both leading to the activation of effector caspases and subsequent DNA fragmentation, plasma membrane blebbing and cell shrinkage [11]. The apoptotic extrinsic pathway is induced upon the activation of death receptors on the cell surface [12]. Conversely, the intrinsic pathway can be activated through various signals such as DNA damage, reactive oxygen species (ROS) or growth factor deprivation [11]. Following mitochondrial outer membrane permeabilization (MOMP), cytochrome *c* is released into the cytosol where it engages the formation of the apoptosome that will first activate the initiator caspase-9 and then the effector caspases-3 and -7. These two pathways are controlled by the BCL-2 family members: the pro-apoptotic (BAX, BAK, BID or BAD) and the anti-apoptotic proteins (BCL-2, BCL-xL, BCL-w, MCL-1) [11,13,14].

Cancer cells commonly share the ability to escape from programmed cell death. This evasion allows cancer cells to grow and develop into a tumor, while also contributing to treatment resistance [15]. The blockade of cell death is a frequent cause of treatment resistance in GBM [15,16,17,18].

Escape from apoptosis commonly occurs through dysregulation of the pro- and anti-apoptotic proteins in human cancer cells. The overexpression of anti-apoptotic proteins at the transcriptional and protein levels has been observed in various cancers. BCL-2 was first described to be constitutively expressed in follicular lymphoma, and the amplification of *MCL1* and *BCL2L1* (encoding BCL-xL) are the most frequent in solid cancers [13]. In GBM, MCL-1 is also overexpressed while high BCL-xL expression is often associated with poor prognosis and advanced disease [19,20]. Additionally, BCL-xL expression was shown to increase with chemotherapy and ionizing radiation in lung cancer. Its role in stemness and aggressiveness is documented in melanoma and GBM [21]. Recently, BH3 mimetics developed to functionally mimic pro-apoptotic BCL-2 proteins were shown to neutralize anti-apoptotic proteins, enabling efficient apoptosis in cancer cells [22].

Considering its important function in regulating the apoptotic response in several cancers, we therefore focused on characterizing the expression and possible role of BCL-xL in GSC growth and possible resistance to BH3 mimetics. The main finding of this short investigative work is that BCL-xL is highly expressed in tumorspheres originating from several GBM cells, rendering them specifically sensitive to BCL-xL inhibition. Therefore, this study provides interesting preliminary data for future research into repurposing BH3 mimetics for GBM treatment.

## 2. Results

### 2.1. High Level of Diversity in BCL-xL Expression in Tumorspheres Compared to Differentiated GBM Cells

**Figure 1:**
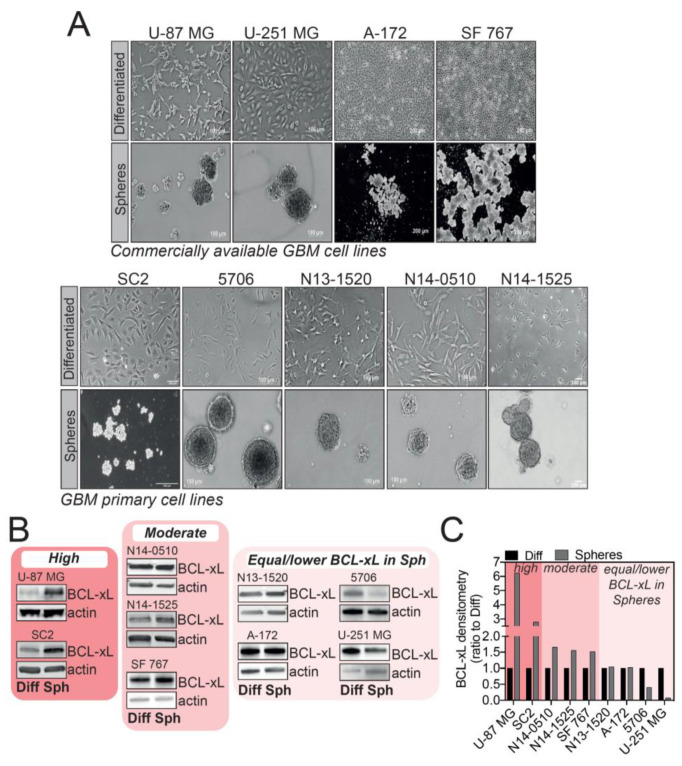
Evaluation of BCL-xL expression in tumorspheres versus differentiated cells in various commercially available and glioblastoma (GBM) patient-derived cell lines. (**A**) Images of GBM cell lines cultured as differentiated cells or tumorspheres. Magnification: 2.5X – 5X. (**B**) Western blot analysis of BCL-xL expression in commercially available and GBM patient-derived cell lines. Full-length blots are presented in Figure S4. (**C**) Densitometry analysis of BCL-xL expression in tumorspheres (ratio to differentiated cells) distinguishing three categories of BCL-xL expression: high, moderate and equal or lower BCL-xL expression in GBM tumorspheres.

As several research articles highlighted a link between resistance to apoptosis and cancer development in GBM, we speculated that anti-apoptotic proteins could be involved in GSC-mediated therapy resistance. Several protocols to isolate and culture GSCs have been described and are currently used; however, the most common one is culturing GBM cells in serum-free medium, complemented with EGF and bFGF, which favors GBM tumorsphere formation (Figure 1A).

We sought to investigate BCL-xL expression in tumorspheres versus differentiated cells using several GBM cell lines, either commercially available or patient-derived GSC tumorsphere cultures. These cells were either grown as adherent, differentiated cells, or allowed to form tumorspheres when deprived of serum (Figure 1A). Given the widely accepted cellular heterogeneity between GBM subtypes and even within the same tumor, it came as no surprise that the different models of cells we tested displayed a different pattern of BCL-xL expression in tumorspheres (Figure 1B). By densitometry analysis, we distinguished three ranges of BCL-xL expression in GBM tumorspheres compared to differentiated cells: high BCL-xL expression (U-87 MG and SC2), moderate BCL-xL increase in expression (N14-0510, N14-1525 and SF 767) and lastly, a group of GBM cells displaying no up-regulation of BCL-xL levels in tumorspheres compared to differentiated cells (N13-1520, A-172, 5706 and U-251 MG). This result highlights that the complexity of GBM is also mirrored in the different patterns of BCL-xL expression in GBM tumorspheres. Of interest, U-87 MG and SC2 cells showed a clear increase in BCL-xL expression when grown as tumorspheres (Figure 1B,C). We thus focused on these cell lines as a model to further investigate the functions of BCL-xL in GBM tumorspheres.

### 2.2. U-87 MG and SC2 GBM Cell-Derived Tumorspheres Upregulate the Anti-Apoptotic Protein BCL-xL

**Figure 2:**
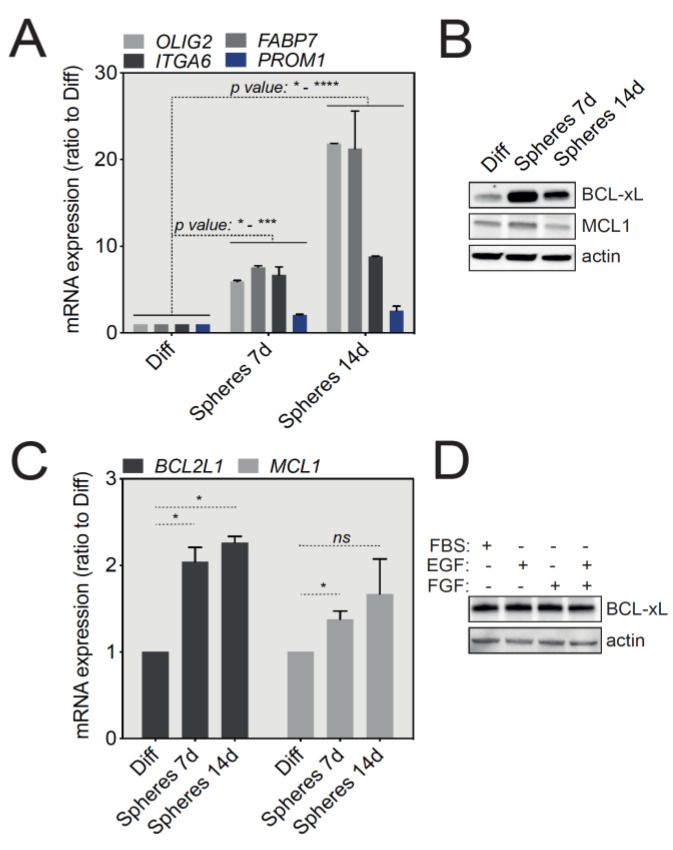
BCL-xL is highly expressed in U-87 MG-derived tumorspheres compared to differentiated cells. (**A**) qRT-PCR analysis of selected GSCs signature markers (OLIG2, ITGA6, FABP7, PROM1) in U-87 MG cells, grown as differentiated or tumorspheres (data represent mean with SEM from three independent experiments, one-way ANOVA, * *p* ≤ 0.05, *** *p* ≤ 0.001, **** *p* ≤ 0.0001). (**B**) Western blot analysis of BCL-xL and MCL-1 expression in U-87 MG cells (differentiated versus tumorspheres). Full-length blots are presented in Figure S4. (**C**) qRT-PCR analysis of *BCL2L1* and *MCL1* mRNA expression in differentiated cells and tumorspheres grown from U-87 MG cells at 7 and 14 days in culture (data represent mean with SEM from three independent experiments, one-way ANOVA, ns *p* > 0.05, * *p* ≤ 0.05). (**D**) Western blot analysis of BCL-xL to test the influence of various growth factors used to grow tumorspheres. Full-length blots are presented in Figure S4.

U-87 MG cells, widely used for in vitro GBM studies, were grown either in adherent standard conditions or in stem cell medium (serum-free, supplemented with EGF and bFGF) and tumorspheres were collected after 7 and 14 days of culture. We first assessed the levels of mRNA expression of several cell lineage-specific proteins enriched in GSCs, including OLIG2, ITGA6, FABP7 and CD133 (*PROM1* gene) by qRT-PCR. As shown in Figure 2A, U-87 MG-derived tumorspheres revealed an upregulation of all the above-mentioned stemness markers that was directly correlated to the duration of tumorsphere culture, compared with adherent cells cultured in the presence of serum. These initial results thus suggest that U-87 MG tumorspheres support the growth of cells with a GSC-like molecular signature.

Since our initial aim was to assess the level of expression of anti-apoptotic proteins in GBM tumorspheres, we next analyzed by Western blot the expression of the major anti-apoptotic proteins, BCL-xL and MCL-1. Importantly, Figure 2B shows a specific and significant upregulation of BCL-xL at the protein level in 7- and 14-day-old tumorspheres compared with differentiated U-87 MG cells, while MCL1 protein levels were not significantly changed. A similar trend in BCL-xL expression was also observed in tumorspheres derived from SC2 GBM primary cells (Figure S1A). BCL-xL protein upregulation in tumorspheres was also mirrored at the transcript level by an increase in *BCL2L1* mRNA, as assessed by qRT-PCR (Figure 2C). To exclude an indirect impact of EGF and bFGF present in tumorsphere cultures, we tested the effect of these cytokines on the expression on BCL-xL. Briefly, U-87 MG cells were cultured in complete medium in the presence of serum and EGF or bFGF, for 24 h. No effect on BCL-xL protein expression was noticed (Figure 2D). Taken together, these results show that GSC-enriched U-87 MG and SC2-derived tumorspheres highly express the anti-apoptotic BCL-xL protein.

### 2.3. BCL-xL Regulates the Size of U-87 MG Tumorspheres

**Figure 3:**
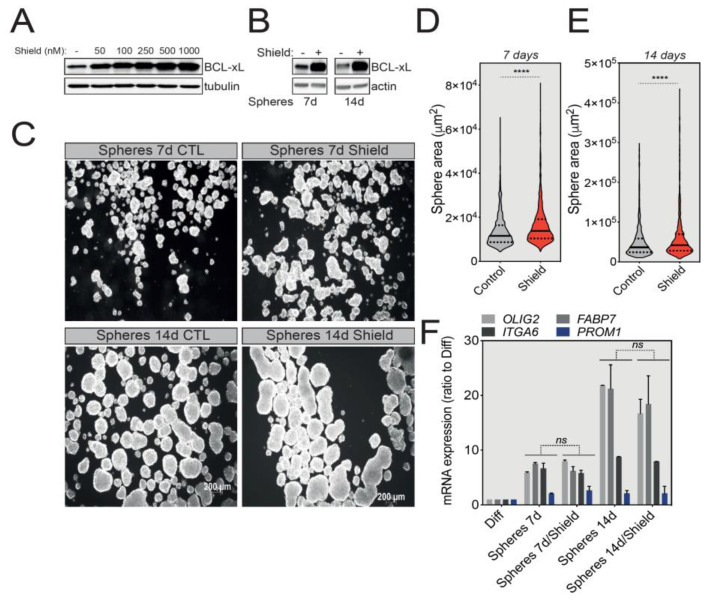
BCL-xL overexpression increases the size of U-87 MG-derived tumorspheres. (**A**) Western blot analysis correlating the expression of BCL-xL with the concentration of Shield-1 ligand used to treat U-87 MG BCL-xL DD cells for 24 h. Full-length blots are presented in Figure S4. (**B**) Western blot analysis of BCL-xL overexpression in U-87 MG tumorspheres after 7 and 14 days of Shield-1 treatment. Full-length blots are presented in Figure S4. (**C**) Representative pictures of U-87 MG tumorspheres following 7 and 14 days of culture in the presence of Shield-1. Magnification: 2.5X. (**D**,**E**) Comparison of the size of U-87 MG tumorspheres after 7 (**D**) or 14 (**E**) days of culture with and without Shield-1. The solid and dotted lines represent the median and the quartile, respectively, **** *p* ≤ 0.0001. (**F**) qRT-PCR analysis of the expression of GSC signature markers (OLIG2, ITGA6, FABP7, PROM1) in U-87 MG cells cultured in the absence or presence of Shield-1 (data represent mean with SEM from three independent experiments, one-way ANOVA, ns *p* > 0.05). The GSC mRNA signatures from untreated tumorspheres correspond to data described in Figure 2.

We next investigated the possible roles that BCL-xL might play in the biology of GBM tumorspheres. For this, we first took advantage of U-87 MG cells stably expressing a degradation-sensitive DD-FLAG-BCL-xL that is therefore constantly degraded in the absence of Shield-1 [23]. To finely tune the overexpression of BCL-xL in spheres, we first tested various concentrations of Shield-1 and choose a working concentration of 100 nM to induce a significant BCL-xL protein accumulation for future experiments (Figure 3A). Next, we treated U-87 MG-derived tumorspheres with Shield-1 for 7 or 14 days (Figure 3B), and then quantified tumorsphere size as an indicator of their growth capacity. As depicted in Figure 3C and confirmed by size quantification (tumorspheres average area) in Figure 3D,E, BCL-xL overexpression significantly increased the size of U-87 MG tumorspheres. Intriguingly, the increased expression of BCL-xL did not affect the stemness markers (OLIG2, ITGA6, FABP7 and PROM1) (Figure 3F).

**Figure 4:**
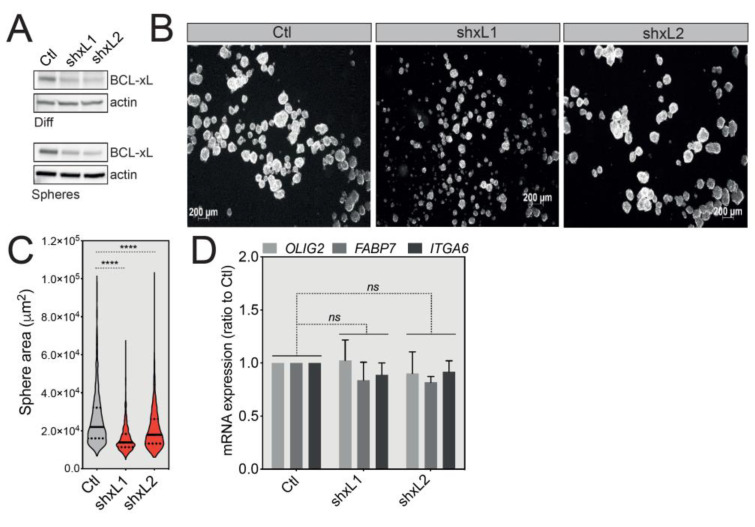
BCL-xL knockdown reduces GBM tumorsphere size. (**A**) Western blot analysis of BCL-xL expression in differentiated or U-87 MG-derived tumorspheres following shRNA-mediated BCL-xL knock-down. Two different specific shRNAs resulted in the same silencing efficacy. Full-length blots are presented in Figure S4. (**B**) Representative images of U-87 MG tumorspheres after BCL-xL silencing. Magnification: 2.5X. (**C**) Comparison of the size of U-87 MG tumorspheres before and after BCL-xL knockdown. The solid and dotted lines represent the median and the quartile, respectively, **** *p* ≤ 0.0001. (**D**) qRT-PCR analysis of the expression of GSC signature markers (OLIG2, FABP7, ITGA6) in control cells versus BCL-xL knockdown tumorspheres (data represent the mean with SEM from three independent experiments, one-way ANOVA, ns *p* > 0.05).

Since GBM spheres are composed of a mixture of GSCs and more differentiated cells, we speculated that BCL-xL might impact the proliferation of the latter cell population. We thus tested the effect of BCL-xL downregulation by stably expressing two lentiviral vectors expressing *BCL2L1*-targeting shRNAs. The efficacy of BCL-xL knockdown was initially validated both in differentiated cells and U-87 MG tumorspheres (Figure 4A). Importantly, BCL-xL knockdown did not affect cell proliferation in the differentiated growth conditions (Figure S2A), while it clearly sensitized U-87 MG cells to actinomycin D-induced apoptosis (Figure S2B). Actinomycin D is a chemotherapeutic drug blocking DNA transcription and therefore inducing apoptosis. Interestingly, knockdown of BCL-xL had the opposite effect on U-87 MG tumorsphere growth compared to its overexpression—namely, the size of tumorspheres was significantly decreased (Figure 4B,C), again without a noticeable effect on the expression of stemness markers (Figure 4D). In summary, these results suggest that in a model of U-87 MG tumorsphere formation, BCL-xL plays a stemness-independent role in the modulation of GBM tumorsphere size.

### 2.4. BCL-xL Upregulation Sensitizes GBM Tumorspheres to BH3 Mimetics-Induced Cell Death

**Figure 5:**
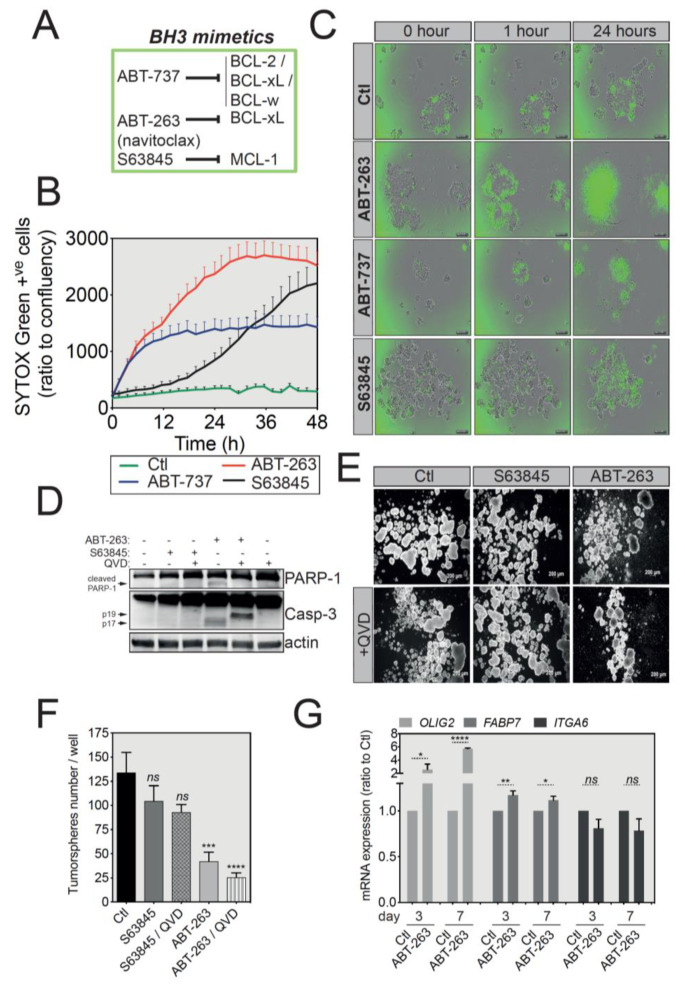
U-87 MG tumorspheres have an increased sensitivity to ABT-263-induced apoptosis. (**A**) Brief summary of the main BH3 mimetics and their preferential targets. (**B**) IncuCyte imager-based cell death induction analysis of U-87 MG-derived tumorspheres treated with ABT-737, ABT-263 or S63845 as indicated. Induction of apoptosis was assessed by SYTOX Green incorporation into permeabilized dead cells. (**C**) Representative pictures of U-87 MG tumorspheres treated with different BH3 mimetics, while the green signal indicates SYTOX Green-positive apoptotic cells Magnification: 4X. (**D**) Western blot analysis of PARP-1 and caspase-3 protein expression and cleavage following treatment with BH3 mimetics. Full-length blots are presented in Figure S4. (**E**) Representative images of U-87 MG tumorspheres in a long-term survival assay. Briefly, following treatment with the indicated BH3 mimetics, U-87 MG-derived tumorspheres were cultured in fresh medium for another week and imaged. Magnification: 2.5X. (**F**) Quantification of tumorsphere number for the long-term survival assay described in (**E**) (data represent mean with SEM from three independent experiments, one-way ANOVA, ns *p* > 0.05, *** *p* ≤ 0.001, **** *p* ≤ 0.0001). (**G**) qRT-PCR analysis for the GSC signature of U-87 MG tumorspheres treated with ATB-263 (data represent mean with SEM from three independent experiments, one-way ANOVA, ns *p* > 0.05, * *p* ≤ 0.05, ** *p* ≤ 0.01, **** *p* ≤ 0.0001).

BH3 mimetics are a novel class of drugs tailored to mimic the function of BH3-only proteins and therefore target the pro-survival members of the BCL-2 family [22]. Several BH3 mimetics are available, each displaying a different level of affinity for a specific anti-apoptotic protein (Figure 5A). To test whether the increased expression of BCL-xL in U-87 MG tumorspheres compared to differentiated cells underlies a newly acquired cell death sensitization to a specific BH3 mimetic, we treated these tumorspheres with the ABT-263, ABT-737 or S63845 BH3 mimetics and assessed the induction of cell death using the IncuCyte live cell imaging coupled with SYTOX Green staining of apoptotic cells. Of note here, caution should be taken when using SYTOX Green staining to assess chemotoxicity. Indeed, the incorporation of this large dye is mediated by the loss of cell membrane integrity that is generally associated with apoptotic cell death. However, in certain cases, the loss of membrane integrity can be transient [24,25]. As shown in Figure 5B,C, U-87 MG tumorspheres underwent apoptosis much faster (as early as one hour) and at a much higher rate when treated with ABT-263 and ABT-737, two of the BH3 mimetics targeting BCL-xL and BCL2/BCL-xL/BCL-w, respectively. Designed to inhibit both BCL-xL and BCL-2, ABT-263 has a higher pro-apoptotic efficacy in cancers overexpressing BCL-xL [26,27]. Of interest, the MCL-1 inhibitor, S63845, had a lower killing efficacy (Figure 5B,C). A similar effect was also observed for the primary GBM cells SC2 (Figure S3A). In addition, a short treatment of only 3 h with ABT-263, but not S63845, efficiently activated the effector caspase-3, as shown by caspase-3 processing and PARP-1 cleavage in U-87 MG cells (Figure 5D and Figure S3B). In SC2-derived tumorspheres, both ABT-263 and S63845 trigger caspase-3 activation and PARP-1 cleavage, yet to a lesser extent for the MCL-1 inhibitor. Interestingly, the pan-caspase inhibitor Q-VD-OPh blocked the processing of caspase-3 subunit p19 into the completely active p17 subunit, therefore confirming that ABT-263 triggers a caspase-dependent cell death in U-87 MG tumorspheres (Figure 5D). We also performed a long-term tumorsphere survival assay, in which U-87 MG spheres were briefly treated for 3 h with either ABT-263 or S63845 and then allowed to grow in fresh medium for 7 days. As depicted in the representative sphere pictures and in corresponding quantifications, the spheres obtained following ABT-263 treatment were both smaller in size (Figure 5E) and fewer in number (Figure 5F) than both the control and spheres treated with the MCL-1 inhibitor, indicating the long-term effect of this BH3 mimetic. Moreover, in this long-term survival assay, Q-VD-OPh did not rescue tumorsphere growth, exposing mitochondrial outer membrane permeabilization as the point of no return for tumorsphere cell apoptosis. To assess whether ABT-263 treatment affected the GSC population, we conducted a qRT-PCR assay on tumorspheres treated with ABT-263 and observed that while there was no change for ITGA6, OLIG2 and FABP7 expression increased significantly (Figure 5G). Collectively, these results provide important insights into targeting GBM tumorspheres using tailored BH3 mimetics, owing to their elevated anti-apoptotic expression.

## 3. Discussion

In this report, we investigated the pattern of BCL-xL expression and its possible functions in GBM tumorspheres, especially in U-87 MG and patient-derived SC2 cells. Compared to differentiated cells, U-87 MG and SC2 tumorspheres express high levels of BCL-xL at both transcript and protein levels, independently of medium composition, suggesting an important role for BCL-xL in tumorsphere formation. Indeed, by artificially increasing BCL-xL expression in U-87 MG tumorspheres, we observed a significant increase in their size. Conversely, this was reverted when BCL-xL was downregulated via shRNA, and the latter cells were extremely sensitive to actinomycin D, which is highly reminiscent of results obtained with MCL-1 knockdown in GBM cells exposed to BH3 mimetics [19]. A limitation of this study might be the use of long-term established cell lines that do not recapitulate all the characteristics of freshly isolated tumor cells from GBM samples [28]. Yet, we also used several patient-derived GBM cells, continuously cultured in well-defined nondifferentiation conditions.

As confirmed by the upregulation of stemness genes, the population of GSCs is clearly more abundant in U-87 MG tumorspheres compared to adherent cells. However, we cannot prove at this stage that BCL-xL is specifically upregulated in the GSC compartment, given that tumorspheres are certainly composed of a mix of GSCs, progenitors and differentiated cells [29]. To address this issue in future experiments, we would need to isolate the two populations (differentiated and GSCs) from tumorspheres and compare their respective expression of BCL-xL. This could be achieved, for instance, via flow cytometry-based cell sorting according to the expression of a cell surface marker specifically expressed by GSCs. Although commonly described as a marker of GSCs, CD133 is probably not the most appropriate stemness marker from our point of view and according to other studies [30,31,32]. However, OLIG2 and ITGA6 appear to be promising alternatives to faithfully characterize U87-MG GSCs, with ITGA6 being a possible plasma membrane marker that could allow cell sorting of GBM stem cells [33]. In line with our findings, Trisciuoglio and colleagues observed an increase in the number of tumorspheres when BCL-xL is overexpressed in GBM cells, while the opposite was noticed when using BCL-xL depleted cells [21]. Although they primarily focused on demonstrating that exogenous BCL-xL controls several hallmarks of cancer aggressiveness such as migration, invasion and tumor cell plasticity, both in GBM and melanoma, these authors also noted that BCL-xL overexpression positively regulated the cancer stem cell phenotype in tumorspheres compared to control cells [21].

To improve our understanding of the transcriptional and/or translational regulation of BCL-xL in GBM tumorspheres, an extensive study of various signaling pathways should be envisaged. Since BCL-xL can be regulated by a plethora of transcription factors ranging from STAT to Rel/NFkB or ETS, further insights into the mode of action of these regulators may unravel the specific regulation of BCL-xL in GBM tumorspheres [34]. Interestingly, when using the Verhaak et al. molecular subtype classification, U-87 MG and SC2 cells presenting the highest BCL-xL expression when cultured as tumorspheres belong to the mesenchymal subtype [35]. According to this study, the NFkB pathway is highly activated in this subtype, which can explain BCL-xL upregulation [35].

A growing body of evidence suggests apoptosis-independent functions for several BCL2 family members, ranging from cell cycle to DNA damage response, metabolism or autophagy [36]. In this article we focused on the anti-apoptotic function of BCL-xL and more specifically on revealing a therapeutic sensitivity to BH3 mimetics targeting BCL-xL. Indeed, the upregulation of BCL-xL in U-87 MG- and SC2-derived tumorspheres compared to differentiated cells is linked to a higher sensitivity to BCL-xL-specific BH3 mimetics. This finding corroborates a study by Liwak and colleagues that reported higher sensitivity of GBM cells to a combination of ABT-737 and doxorubicin, owing to increased levels of BCL-xL in a specific subset of glioblastoma cell lines [37]. This is consistent with earlier pioneering work that advocated the use of BH3 mimetics for triggering apoptosis in GBM cells, alone or in combination with ionizing irradiation or temozolomide [38,39,40]. As mentioned in the results section, we characterized the chemosensitivity of GBM cells to BH3 mimetics by using SYTOX Green incorporation and live cell microscopy. Since this fluorescent large dye can also be incorporated by cells undergoing transient plasma membrane opening, to what extent our measurements of cytotoxicity reflect cell death versus reversible loss of cell membrane integrity remains to be determined [24,25].

We present here promising findings that highlight means of killing GSCs, responsible for GBM recurrence. However, these results should be interpreted with caution as our findings are neither applicable to all patient-derived nor commercially available GBM cell lines. Moreover, our findings will need to be validated in GBM mouse models and ideally on GSCs derived from human GBM biopsies. Furthermore, as shown in Figure 5G, triggering apoptosis in GBM tumorspheres increases the expression of certain stemness markers (OLIG2 and FABP7). This might be due to a pro-mitogenic paracrine effect of apoptotic cells on neighboring GSCs, as part of a wound healing and tissue regeneration reaction [41]. To be clinically pertinent, the use of BH3 mimetics for GBM treatment will need to be conditioned by identifying the appropriate populations of patients that would benefit from this treatment. The newly described high-throughput dynamic BH3 profiling might be helpful in identifying dependencies on certain anti-apoptotic proteins in fresh GBM samples and make an informed therapeutic choice for the right BH3 mimetic [42]. Integrating systems biology into clinical practice can also help in predicting treatment response in GBM patients [43].

BCL-xL can be targeted only if fresh tumor samples grown in tumorsphere permissive medium exhibit higher BCL-xL expression. Nevertheless, the use of BH3 mimetics for GBM treatment could alleviate other pro-oncogenic effects of BCL-xL. For instance, BCL-xL was shown to function as an epigenetic modifier and promote both epithelial to mesenchymal transition (EMT) and stemness in pancreatic, breast and lung cancer [44,45]. This is also consistent with a growing number of pre-clinical studies confirming the benefits of using BH3-mimetics when treating gliomas [46].

## 4. Materials and Methods

### 4.1. Cell Lines

The U-87 MG, U-251 MG, A-172, SF 767 human glioblastoma cell lines were obtained from the American Type Culture Collection (ATCC). The N13-1520, N14-0510, N14-1525, 5706 and SC2 are patient-derived cell lines obtained from M. Gabut’s laboratory (CRCL). These primary cells were maintained as tumorspheres in stem cell medium (SCM) composed of DMEM F12 (ThermoFisher Scientific, 31331-093, Waltham, MA, USA), B-27™ supplement 1× (Life Technologies, 17504044, Carlsbad, CA, USA), EGF 20 ng/mL, recombinant human basic FGF 10 ng/mL (Peprotech, 100-18B, Neuilly-Sur-Seine, France) and 1% penicillin/streptomycin. For differentiation, cells were cultured for at least 7 days in DMEM (differentiation media) supplemented with 2 mM L-glutamine (ThermoFisher Scientific, 25030-24), non-essential amino acids (ThermoFisher Scientific, 11140-035), 1 mM sodium pyruvate (ThermoFisher Scientific, 11360-039), 10% FBS (Eurobio, CVFSVF00-01, Les Ulis, France) and 1% penicillin/streptomycin (ThermoFisher Scientific, 15140-122). The U-87 MG, U-251 MG, A-172, and SF 767 were grown in differentiation medium and for obtaining tumorspheres, they were cultured for at least 7 days in SCM medium. Cells formed tumorspheres only in plates with ultra-low attachment surface. In order to work in clonal conditions, 5 × 10^3^ cells were grown in 2 mL of SCM medium in a 6-well plate. Cells were regularly checked for mycoplasma contamination.

### 4.2. Reagents

Q-VD-OPh (Clinisciences, JM-1170, Nanterre, France), SYTOX Green (ThermoFisher Scientific, S34860), Actinomycin D (Sigma, A9415, Saint-Louis, MO, USA), ABT737 (Clinisciences, A8193), ABT263 and S63845 (Clinisciences, A8737) were employed in this study.

### 4.3. Stable Cell Line Generation

After plating Phoenix Ampho 293T cells at a cell density of 1.5 × 10^6^ in a 10 cm dish, these cells were transfected 24 h later with the retroviral constructs (pLZRS DD-FLAG-BCL-xL and pSuper.retro-shRNA BCL-xL) using Lipofectamine 2000 (Invitrogen). One day after transfection, in order to recover the viruses, the supernatant was harvested, filtered, supplemented with polybrene (1 µg/mL) and used to infect U-87 MG cells. Forty-eight hours post-infection, the selection of the cells stably expressing DD-FLAG-BCL-xL was achieved using Zeocin (200 µg/mL, Invivogen) or puromycin (1 µg/mL). The sequence of shRNA BCL-xL 1 (or shxL1) is ACCAGGAGAACCACTACATGCAGCC, while that of shRNA BCL-xL 2 (or shxL2) is GTTCCAGCTCTTTGAAATAGTCTGT.

### 4.4. Western Blotting

Cell lysates were prepared using RIPA lysis buffer (Cell Signaling, 9806S) supplemented with phosphatase inhibitors complex 2 and 3 (Sigma Aldrich, P5726-1ML, P6044-1ML), DTT 10 mM and protease inhibitor cocktail (Sigma-Aldrich, 4693116001). Protein concentration was assessed using the Protein Assay dye Reagent Concentrate (Biorad, 50000006). Equal amounts (20 µg) of each sample were separated on 4–12% SDS-polyacrylamide gels (Biorad) under denaturating conditions (SDS PAGE Sample loading buffer (VWR, GENO786-701, Radnor, PA, USA) supplemented with 1 mM DTT) and transferred onto a nitrocellulose membrane using the Transblot Turbo Transfer System (Biorad, 1704150EDU). Non-specific binding sites were blocked for 1 h with 5% dry milk or BSA in TBS-Tween 0.1%, while the primary antibody (1/1000 in 1% BSA TBS-Tween 0.1%) was incubated overnight at 4 °C, under agitation. The following primary antibodies were used: actin (Sigma-Aldrich, A3854), BCL-xL (Cell Signaling, 2764S), MCL-1 (Cell Signaling, 4572S), PARP-1 (Cell Signaling, 9532), caspase-3 (Cell Signaling, 9662S) and HSC70 (Santa Cruz, sc-7298). The nitrocellulose membranes were then rinsed three times in TBST 0.1% then incubated with appropriate HRP-coupled secondary antibody (Biorad, 1706515, 1706516; 1/5000) for 1 h at room temperature. Three extra washing steps were performed. The detection of the antibodies was achieved by chemiluminescence (Clarity Western ECL reagent, Biorad, 1705060) according to the manufacturer’s instructions and using chemiDoc Imager (Biorad, 17001401).

### 4.5. Quantitative RT-PCR

Total RNA was extracted using the Nucleospin RNA protocol from Macherey-Nagel kit (740955, Hoerdt, France), while the cDNA synthesis was done using the Sensifast cDNA kit (Bioline, BIO-65053). cDNAs were then amplified by PCR using specific primers for each gene, designed using the Primer-blast software (https://www.ncbi.nlm.nih.gov/tools/primer-blast/) and listed in Table 1. GAPDH and ACTB are housekeeping genes. PCR fragments were amplified at 95 °C for 2 min, followed by 40 cycles at 95 °C for 5 s and 60 °C for 30 s. For the qRT-PCR experiments we used SensiFAST SYBR No-ROX kit (Bio-Technofix, BIO-98020, Guibeville, France) and a Lightcycler96 machine (Roche, IN, USA).

### 4.6. Apoptosis Assay

Under a binocular microscope and in sterile conditions, 1-2 tumorspheres were plated in each well of a ULA 96-well plate (Greiner) and then treated with various compounds: 10 µM of ABT-737, ABT-263 or S63845, Q-VD-OPh 20 µM or with actinomycin D 1 µM in the presence of 30 nM SYTOX Green (ThermoFisher Scientific, S34860). Cells were then imaged every 60 min using the IncuCyte ZOOM imager.

### 4.7. Image Analysis

Measurements of tumorsphere size were performed using the ImageJ software 1.52a.

### 4.8. Statistical Analysis

Data are expressed as the mean ± SEM. A two-tailed Student’s t-test was applied to compare two groups of data. Analyses were performed using the Prism 5.0 software (GraphPad, San Diego, CA, USA). *p*-values < 0.05 were considered to indicate a statistically significant difference.

## 5. Conclusions

This study reveals a different pattern of expression for the anti-apoptotic protein BCL-xL in human GBM cells. Indeed, U-87 MG and primary GBM SC2 cells show a higher expression of BCL-xL when cultured as tumorspheres, while their size is correlated to BCL-xL protein content. This article also highlights the sensitivity of glioblastoma tumorspheres to BH3 mimetics targeting BCL-xL, suggesting that these drugs could be repurposed for glioblastoma treatment if the status of anti-apoptotic proteins is well characterized in patient samples.

## Figures and Tables

**Table 1:** List of specific primers used for RT-qPCR.

Gene of Interest	Forward Primer (5′-3′)	Reverse Primer (5′-3′)
*BCL2L1*	AAAAGATCTTCCGGGGGCTG	TCTGAAGGGAGAGAAAGAGATTCA
*ACTB*	AGAGCTACGAGCTGCCTGAC	AGCACTGTGTTGGCGTACAG
*OLIG2*	CCTAAAGGTGCGGATGCTTAT	ATCTGGATGCGATTTGAGGAG
*FABP7*	AGCTGACCAACAGTCAGAAC	CCGTTGGTTTGGTCACATTTC
*ITGA6*	TTGGACTCAGGGAAAGGTAT TG	TGCAGACTTCATGTCTCTCTTC
*PROM1*	CCCAACATCATCCCTGTTCTT	CTGCTGCTAAGCTGTGTACTT
*MCL1*	CCAAGAAAGCTGCATCGAACCAT	CAGCACATTCCTGATGCCACCT
*GAPDH*	TGCACCACCAACTGCTTAGC	GGCATGGACTGTGGTCATGAG

## Supplementary Materials

The following are available online at https://www.mdpi.com/2072-6694/12/10/2853/s1, Figure S1, (a) BCL-xL and MCL-1 protein expression in SC2 cells grown as differentiated cells or tumorspheres for 7 days. Figure S2, (a) Cell growth analysis based on IncuCyte live imaging of control U-87 MG and shRNA BCL-xL cells grown in differentiated conditions, (b) Apoptosis induction based on SYTOX Green incorporation following actinomycin D (1 μM) treatment of control U-87 MG and shRNA BCL-xL cells grown in differentiated conditions, Figure S3, (a) IncuCyte imager-based cell death induction analysis of SC2-derived tumorspheres treated with ABT-737, ABT-263 or S63845 as indicated. Induction of apoptosis was assessed by SYTOX Green incorporation into permeabilized dead cells, (b) Western blot analysis of cleaved PARP-1 and caspase-3 following treatment of SC2-derived tumorspheres with BH3 mimetics, Figure S4. Uncropped Western blots.
