# Supplementary material for: Profiling Anti-Apoptotic BCL-xL Protein Expression in Glioblastoma Tumorspheres
Source: Cancers (Basel). 2020 Oct 2;12(10):2853. doi: 10.3390/cancers12102853 (PMC7599739; doi:10.3390/cancers12102853)

Supplementary Figure 1

A

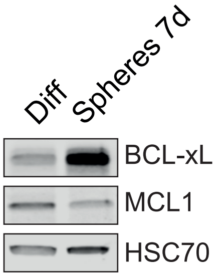

Supplementary Figure 2

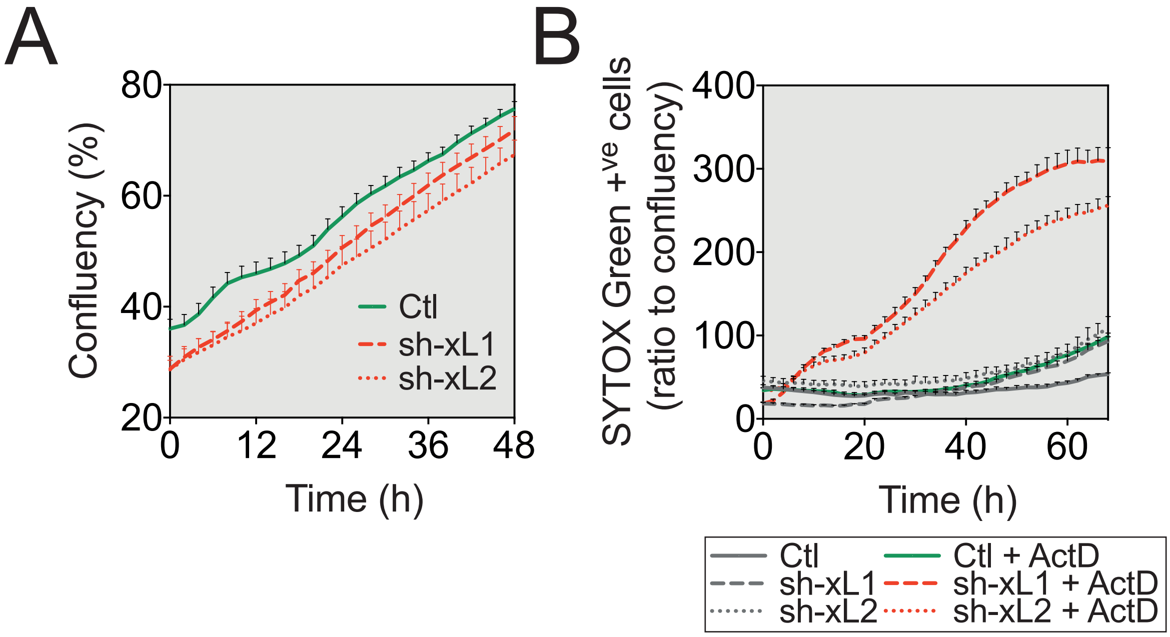

Supplementary Figure 3

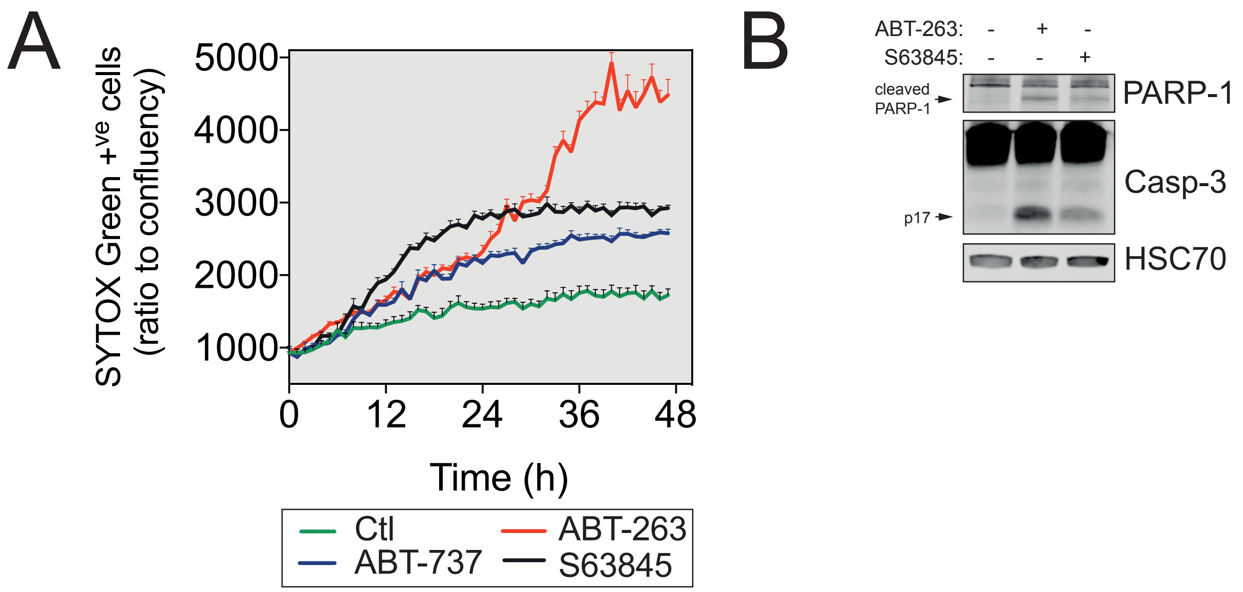

Supplementary Figure 4

A. raw data Figure 1b

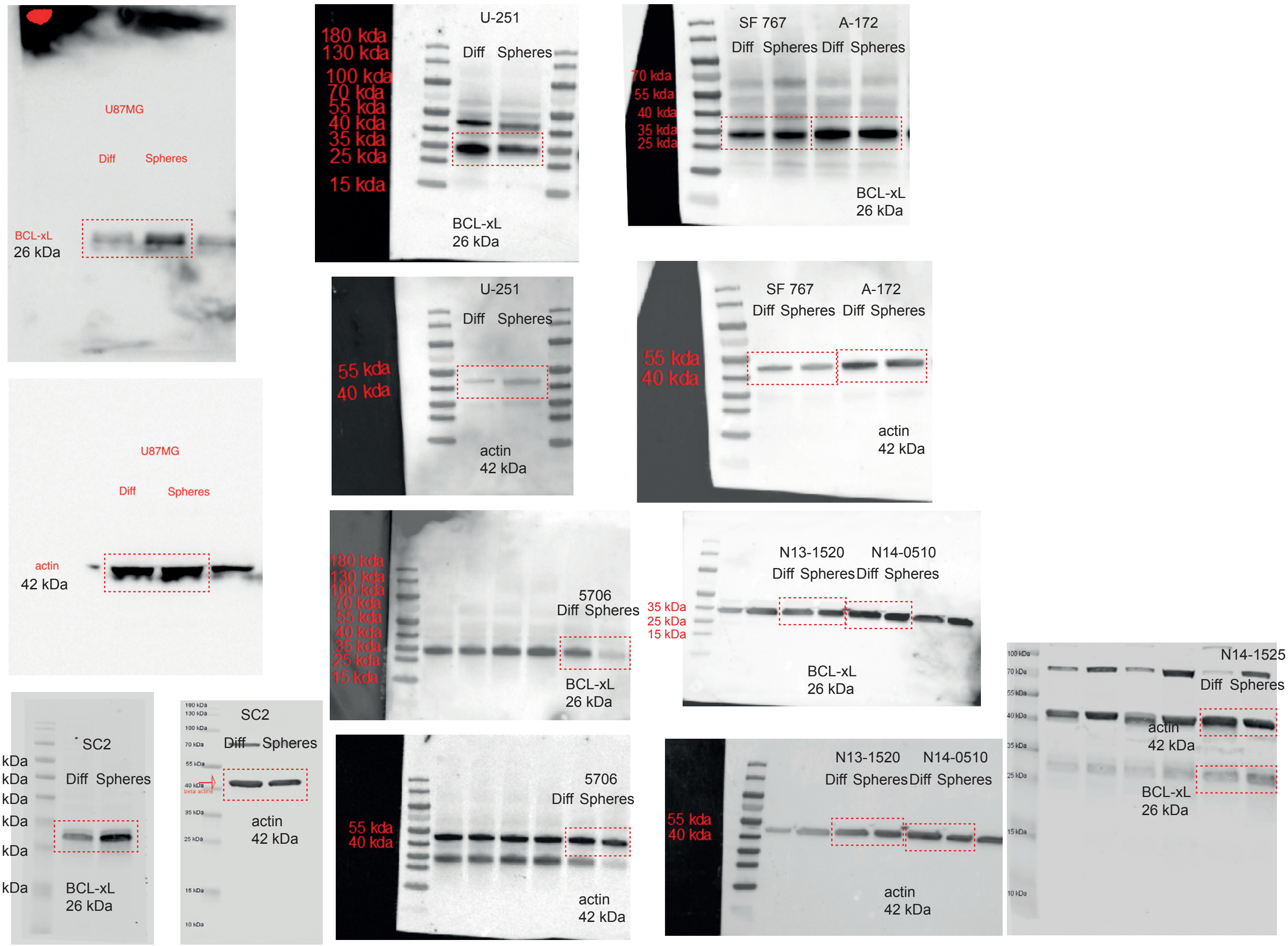

B. raw data Figure 2b

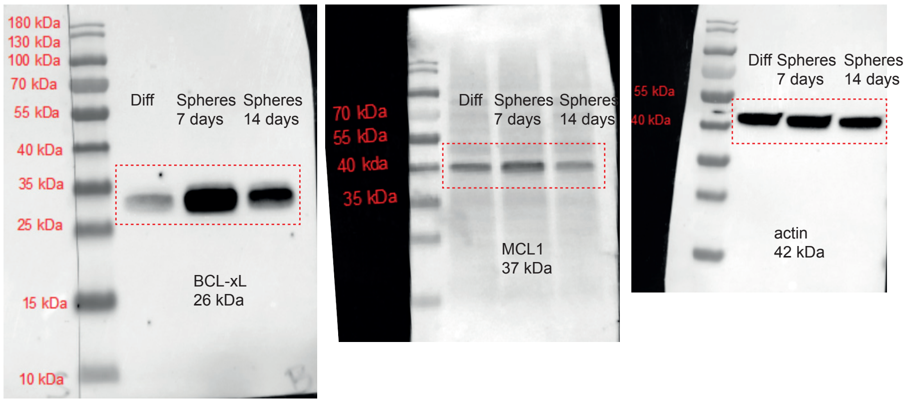

C. raw data Figure 2d

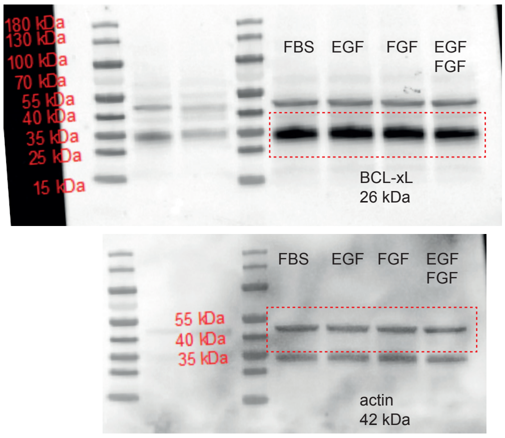

D. raw data Figure 3a

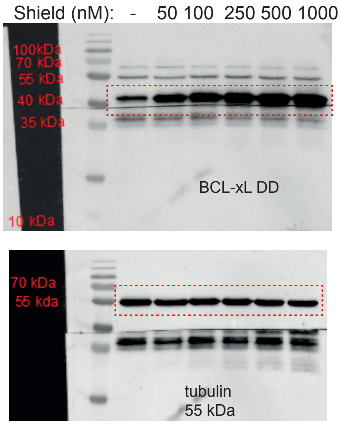

E. raw data Figure 3b

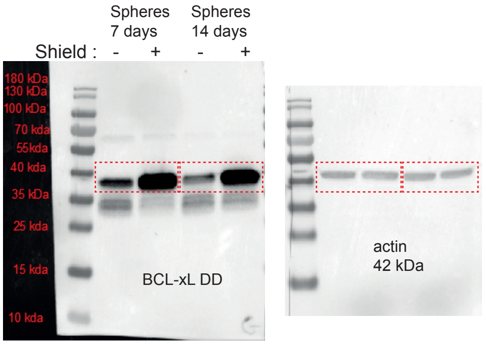

F. raw data Figure 4a

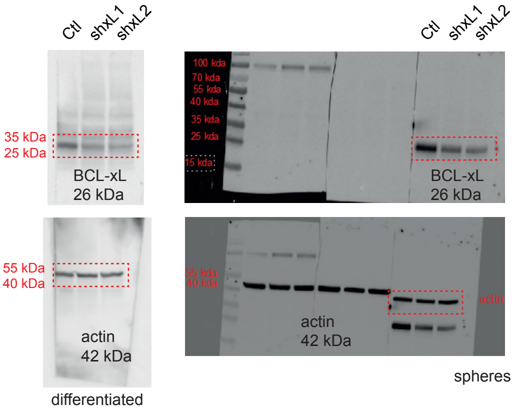

Supplementary Figure 4 (continued)

G. raw data Figure 5d

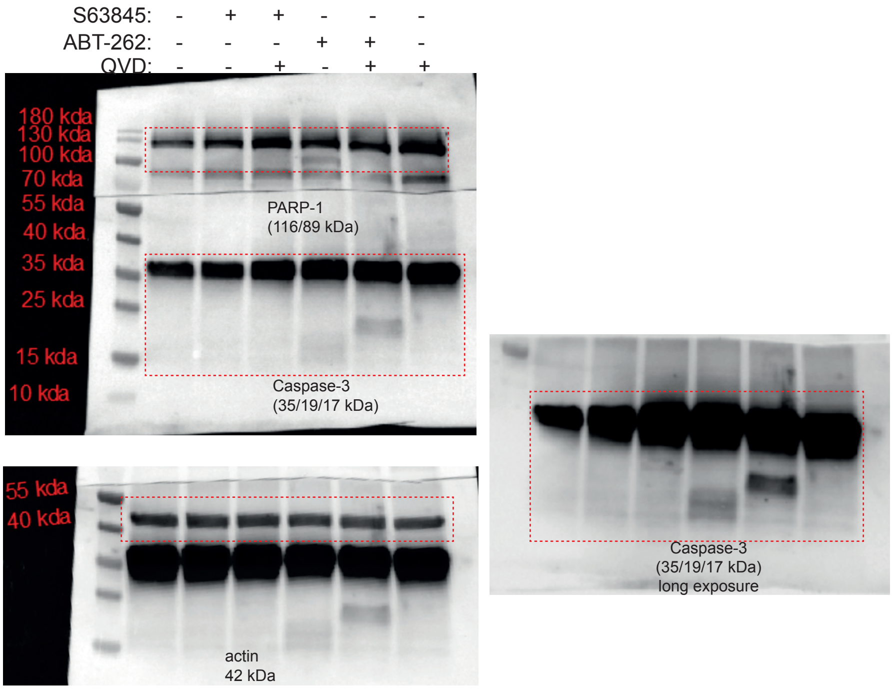

H. raw data Supp Figure 1

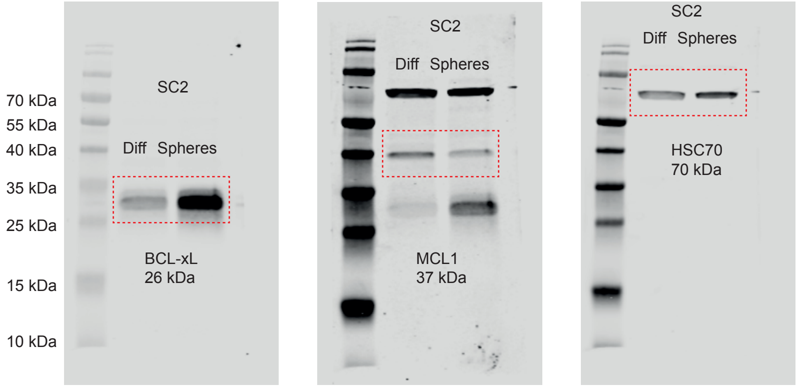

I. raw data Supp Figure 3B

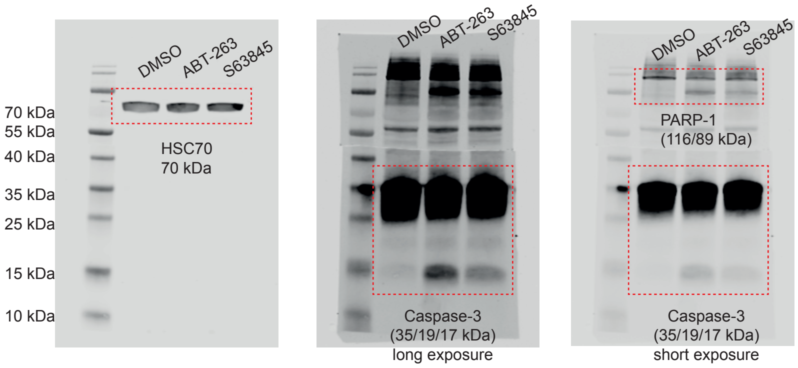

Supplement: Supplementary file 1 [file cancers-12-02853-s001.pdf]
